# Spectral Analysis of Sequence Variability in Basic-Helix-loop-helix (bHLH) Protein Domains

**Published:** 2007-02-09

**Authors:** Zhi Wang, William R. Atchley

**Affiliations:** 1 Graduate Program In Biomathematics And Bioinformatics; 2 Department Of Genetics and Center For Computational Biology, North Carolina State University, Raleigh, NC 27695-7614, USA

**Keywords:** Spectral analysis, bHLH proteins, entropy, factor analysis, molecular architecture

## Abstract

The basic helix-loop-helix (bHLH) family of transcription factors is used as a paradigm to explore structural implications of periodicity patterns in amino acid sequence variability. A Boltzmann-Shannon entropy profile represents site-by-site amino acid variation in the bHLH domain. Spectral analysis of almost 200 bHLH sequences documents the periodic nature of the bHLH sequence variation. Spectral analyses provide strong evidence that the patterns of amino acid variation in large numbers of sequences conform to the classical *α*-helix three-dimensional structure periodicity of 3.6 amino acids per turn. Multivariate indices of amino acid physiochemical attributes derived from almost 500 amino acid attributes are used to provide information regarding the underlying causal components of the bHLH sequence variability. Five multivariate attribute indices are used that reflect patterns in i) polarity - hydrophobicity - accessibility, ii) propensity for secondary structures, iii) molecular volume, iv) codon composition and v) electrostatic charge. Multiple regression analyses of the entropy values as dependent variables and the factor score means and variances as independent variables are used to partition variation in entropy values into their underlying causal structural components.

## Introduction

Contemporary research in biological, medical and agricultural sciences often focuses on the architecture of complex traits. Complex traits are composed of various component parts that are interdependent, dynamic and multi-regulated. Protein molecules are complex traits. They i) contain multiple structural and functional domains that may arise independently from different sources; ii) the domains may be integrated into divergent proteins by domain shuffling; iii) domains are composed of many different amino acid sites having varying degrees of intercorrelation; iv) the various amino acids contribute differentially to structure and function; and v) different domains (and their constituent amino acids) may be subjected to separate selection regimes during evolutionary adaptation. To adequately understand evolution and corresponding structural divergence of proteins requires knowledge of the various component parts, their characteristics, dynamics, integration and divergence.

Herein, we explore the periodicity in patterns of site by site amino acid variation to better understand relationships between sequence diversity and protein structure. Specifically, we ask if observed patterns of within-site variability exhibit a systemic *periodicity* that corresponds to the known structural geometry derived from crystal structure studies. Further, we explore the underlying multidimensional causes of sequence diversity.

A number of authors have suggested that individual amino acids show patterns of periodicity that suggest important characteristics in molecular structure (e.g. Eisenberg et al. 1984; Pasquier et al. 1998; Leonov and Arkin, 2005). For example, an *α*-helix adopts a spiral configuration of 99° ± 7° around the axis, generating a range in periodocity of 3.40–3.91 aa per turn, with an average periodocity of about 3.6 aa per turn (Kyte, 1995). Mutations that disrupt such structural geometry are probably subjected to strong natural selection (Patthy, 1999).

In the present work, we explore the periodic behavior of site by site amino acid variability or *diversity* in a large collection of basic helix-loop-helix proteins (Atchley and Fitch, 1997). bHLH proteins are a collection of important transcriptional regulators involved with the control of a wide variety of developmental processes in eukaryote organisms (Murre et al. 1989, Murre et al. 1994; Sun and Baltimore, 1991; Atchley and Fitch, 1997; Ledent and Vervoort, 2001). Herein, we use spectral analysis, information theory and multivariate statistical methods to: 1) describe periodicity patterns in amino acid diversity within the highly conserved bHLH protein domain; 2) ascertain whether diversity in amino acid composition conforms to estimates of secondary structure shown by crystal studies; and 3) decompose variability in entropy patterns into its underlying structural components.

The present paper is one of a series using methods from computational biology to explore a number of structural and evolutionary aspects of the basic helix-loop-helix (bHLH) family of proteins (e.g. Atchley and Fitch, 1997; Morgenstern and Atchley, 1999; Atchley et al. 2000, Atchley et al. 2001; Wollenberg and Atchley, 2000; Atchley and Fernandes, 2005; Atchley and Buck, 2006).

## Materials and Methods

### Definition and Structure of the bHLH Domain

The bHLH domain is a highly conserved region comprised of approximately 60 amino acids (Atchley and Fitch, 1997). It is best modeled as two separate *α*-helices separated by a variable length loop (Ferre-D’Amare et al. 1993, 1994; Shimizu et al. 1997). The basic (b) DNA binding region of about 14 amino acids interacts with a consensus hexanucleotide E-box (CANNTG). bHLH proteins are classified into 5 major DNA-binding groups (A, B, C, D, and E) based on how the proteins bind to the consensus E-box and other attributes (Atchley and Fitch, 1997; Ledent and Vervoort, 2001). The helix regions (H1 and H2) are involved in protein-DNA contacts and protein-protein interaction, i.e. dimerization. The variable length loop region (L) may range from approximately 5 to 50 residues.

Herein, we analyze variation in 196 bHLH sequences of the bHLH subfamilies and DNA binding groups including 83, 72, 16, 9 and 16 sequences belonging to DNA binding groups A, B, C, D and E, respectively. These sequences are part of a standard bHLH dataset used in a number of previous computational analyses (e.g. Atchley and Fitch, 1997; Atchley et al. 2000; Atchley et al. 2005).

### Data preparation

Sequences were aligned using both local and global type alignment algorithms and the resultant alignments then corrected by eye when the results of the two alignment algorithms did not agree. Representatives of the aligned subfamilies can be found in Atchley and Fitch (1997). The amino acid components of the bHLH domain follow the structural analyses of Ferre-D’Amare et al. (1993): basic region (amino acids 1–13), helix 1 (14–28), loop (29–49), and helix 2 (50–64).

The loop region is highly divergent in both length and amino acid composition making accurate decisions about homology difficult for much of this region (Atchley and Fitch, 1997; Morgenstern and Atchley, 1999). Unless an accurate alignment is obtained, statistical analyses are of dubious value since putatively non-homologous amino acids are being compared. Thus, part of the highly variable interior portion of the loop region was removed and only 49 columns of the multiple alignments remain for spectral and statistical analysis. Removal of the non-homologous portion before subsequent analyses is standard procedure. Preliminary spectral density plots of the profile containing the whole loop region were compared and the results and conclusions were not affected by removing the heterogeneous portion of the loop region.

### Entropy Profiles

**Figure 1:**
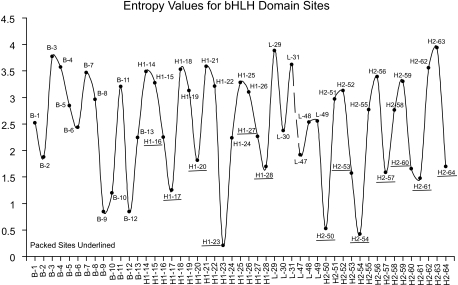
Entropy profile of bHLH protein domains suggesting an oscillation pattern.

**Figure 2:**
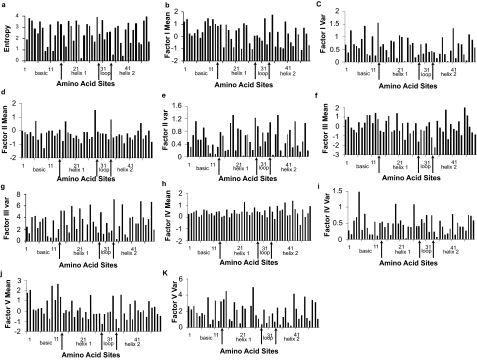
Entropy and Factor profiles of bHLH protein domains. (a) Entropy vs. Amino Acid Sites. (b) Factor I Means vs. Amino Acid Sites. (c) Factor I Variance vs. Amino Acid Sites. (d) Factor II Means vs. Amino Acid Sites. (e) Factor II Variances vs. Amino Acid Sites. (f) Factor III Means vs. Amino Acid Sites. (g) Factor III Variance vs. Amino Acid Sites. (h) Factor IV Means vs. Amino Acid Sites. (i) Factor IV Variances vs. Amino Acid Sites. (j) Factor V Means vs. Amino Acid Sites. (k) Factor V Variances vs. Amino Acid Sites.

The Boltzmann-Shannon entropy *E* is used to quantify sequence variability of amino acid residues at each aligned amino acid site (Atchley et al. 1999, Atchley et al. 2000). It is calculated as *E(p)* = −∑*_j_*_=1_^21^*p_j_* log_2_ (*pj*), where *p_j_* is the probability of a residue being a specific amino acid or a gap, and 0 ≤ *E* (*p*) ≤ 4.39. An “entropy profile” is given in a scatter plot (Fig. 1) and a histogram (Fig. 2a) where the height of the individual bars reflects the entropy value (residue diversity) at a particular aligned amino acid site. Small *E* values indicate a high degree of sequence conservation.

### Factor Score transformations

Statistical analysis of alphabetic sequence data is hindered by the lack a rational underlying metric for alphabetic codes (Atchley et al. 2005). To resolve this “metric” problem, these authors used multivariate statistical analyses of 495 amino acid physiochemical attributes to generate a small set of highly interpretable numerical values that summarize complex patterns of amino acid attribute covariation. Using factor analysis (Johnson and Wichern, 2002), these authors defined five major patterns of amino acid attribute covariation that summarize the most important physiochemical aspects of amino acid covariability. These five patterns or multidimensional indices were interpreted as follows: Factor I = a complex index reflecting highly intercorrelated attributes for polarity, hydrophobicity, and solvent accessibility. Factor II = propensity to form various secondary structures, eg coil, turn or bend versus alpha helix frequency. Factor III = molecular size or volume, including bulkiness, residue volume, average volume of a buried residue, side chain volume, and molecular weight. Factor IV = relative amino acid composition in various proteins, number of codon coding for an amino acid, and amino acid composition. Factor V = electrostatic charge including isoelectric point and net charge. A set of *“factor scores”* arising from these analyses provide a multidimensional index value that positions every amino acid in each of these major interpretable patterns of physiochemical variation.

Herein, we transform the original alphabetic amino acid codes in the aligned bHLH sequence data to these five factor scores. This procedure generates five sets of numerical values that accurately reflect a broad spectrum of amino acid attributes. The factor score transformed data are then used in our statistical analyses. For simplicity, we analyze the five sets of factor score transformed data separately rather than an analysis of all five factors simultaneously.

To better understand the underlying causes of diversity in amino acids, we include analyses of both the factor score means and variances graphed in histograms (Fig. 2. b–k). The former replaces alphabetic data with the average multidimensional amino acid attribute while the latter uses its variability.

### Spectral Analysis Based on Fourier Transformation (FT)

It is well-known that *individual* amino acid sequences can exhibit a periodic pattern in the occurrence of certain types of amino acids (Pasquier et al. 1998). What is not clear is whether site by site amino acid variation computed for large numbers of sequences also exhibit periodic patterns.

**Figure 3:**
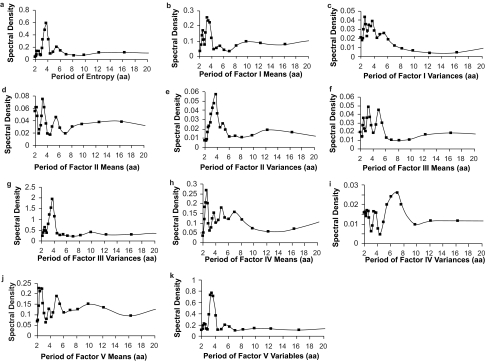
Plots of the spectral density distribution of entropy, Factor score means and variances profiles produced by the Fourier transformation. (a) Spectral density plot of entropy profile. (b) Spectral density plot of Factor I means profile. (c) Spectral density plot of Factor I variances profile. (d) Spectral density plot of Factor II means profile. (e) Spectral density plot of Factor II variances profile. (f) Spectral density plot of Factor III means profile. (g) Spectral density plot of Factor III variances profile. (h) Spectral density plot of Factor IV means profile. (i) Spectral density plot of Factor IV variances profile. (j) Spectral density plot of Factor V means profile. (k) Spectral density plot of Factor V variances profile.

To explore this question, a time series model is applied that is expressed in terms of sine and cosine components (Bloomfield, 1976) as

(1) $$Y_{t}=\sum_{i=1}^{m}(A_{i}\text{cos}\;(\omega_{i}t)+B_{i}\text{sin}\;(\omega_{i}t))+e_{t}$$

where *Y_t_* is the original variable with *n* observations. *m*=*n*/2 if *n* is even*; m*=(*n*−1)/2, if *n* is odd. *ω*_i_ specifies the Fourier frequencies, 2*πi*/*n*, where *i*=1, 2, …, *m. A_i_* and *B_i_* are the amplitude of the sine and cosine components and *e_t_* is the error term. The sum of squares of the *A_i_* and *B_i_* can form periodograms by plotting them against frequency or against wavelength. The periodogram is interpreted as the amount of variation in *Y* at each frequency. If there is a significant sinusoidal component at a given frequency, the amplitude *A* or *B* or both will be large and the periodogram will have a large ordinate at that given frequency. If there is no significant sinusoidal component, then the periodogram will not have large ordinates at any frequencies. A Hamming window is applied to produce the spectral density plots, which is a general smoothing procedure in spectral analysis (Kendall and Ord, 1990). The spectral density plots (Fig. 3) of entropy, factor score means and variances are produced by SAS software (PROC SPECTRA).

### Spectral analysis by the Burg method

The Burg method for spectral analysis is based on the well-known autoregressive (AR) modeling technique for processing time-series data (Marple, 1987; Kay, 1988). An AR model provides a parametric description of time-series data. For a given discrete data sequence *x_i_* for 1 ≤*i* ≤ *n*, the sample at index *i* can be approximated by a linear combination of previous *k* observations of the data sequence by

$$X_{i}={\hat{X}}_{i}+e_{i}=-\sum_{k=1}^{k}{\hat{a}}_{k}\;X_{i-k}+e_{i}$$

where *i* ≥ *k*. With the Burg method, the spectral density of the time series can be described in terms of AR model parameters and the corresponding modeling error variance by

(2) $$\begin{array}{l} {\overset{⌢}{P}}_{AR(f)}=\frac{T{\overset{⌢}{\sigma }}^{2}}{|1+\sum_{i=1}^{p}{\overset{⌢}{a}}_{i}\text{exp}\;(-j2\pi fnT){|}^{2}}\\ j^{2}=-1 \end{array}$$

where *σ̑*^2^ is the estimated modeling error variance, and *T* is the sampling interval.

The Burg method is used here as an alternative to the FT method to calculate the spectral density of the entropy as well as the factor score means and variances. Readers are referred to Marple (1987) for more details about Burg method algorithms. The spectral density plots for entropy, factor score means, and factor score variances produced by Matlab software (version 6.5) are very similar to those in Fig. 3 produced by the FT method.

### Statistical Test

When spectral density plots are graphed, “large” peaks occur, whose statistical significance and accuracy requires validation. Fisher’s test (Warner, 1998) is a conservative method for identifying “major” periodic components. Fisher’s test rejects the null hypothesis if the periodogram contains a test statistic significantly larger than the average value (Brockwell and Davis, 1991; Warner, 1998). The test statistic *g*, gives the proportion of the total variance accounted for by the largest periodogram component.

For the bHLH analyses, the critical values of the proportion of variance in Fisher’s test at *α* = 0.05 level (N = 49) are 0.240, 0.156 and 0.122 for the first, second and third largest periodogram ordinates, respectively. The critical value 0.240 implies that if there are 49 data points in the numeric sequence, then the largest periodogram ordinate must account for more than 24% of the variance to be judged significant at the P = 0.05 level. In the special case of a constant time series (constant numeric sequence in this paper), the *p*-value returned in Fisher’s test is exactly 1 (i.e. the null hypothesis is not rejected). If the largest periodogram ordinate is statistically significant, then we test the second and third largest periodogram ordinates for significance, and so on.

Having obtained the major periodic components, harmonic analysis is used to fit the data with the cyclic components (Warner, 1998). Standard methods from elementary Fourier analysis are used to estimate the parameters A and B. *R*-square (*R*^2^) measures goodness of fit of the predictive model and estimates the percentage of total variance of the observations explained by the analysis. Therefore, with the period estimate from the spectral analysis as a prior, we can search for the best period estimate maximizing the *R*-square in a relative small range and its confidence interval (CI).

For the entropy profile, a bootstrap simulation produced 1000 random samples with replacement from the original bHLH multiple alignments. For each sample, the harmonic analysis is conducted to detect the best period estimate with the largest *R*-square statistics. Assuming the 1000 period estimates have a normal distribution, the 95% confidence interval of the mean can be obtained.

### Underlying causes of sequence diversity

Simply knowing particular patterns of variation exist is not sufficient since it is important to know the underlying causes of sequence diversity. Analysis of variance (ANOVA) was used to relate the variance in the entropy values to the variances for Factors I–V. The null hypothesis is that there are no significance differences between the total variation of the scores for Factor I–V and the error variance, i.e. there is no significant added effect due to any particular physiochemical factor.

Multiple regression analysis is used to explore the underlying causes of amino acid diversity. The dependent variable is the entropy value while the independent variables are the five factor scores. Analyses were carried out using both the factor score means and the factor score variances. Multiple regression was used to estimate *β*_0_, *β*_1_, …, *β*_5_ of the following regression model equation.

(3) $$\begin{array}{l} \text{Entropy}=\beta_{0}+\beta_{1}(\text{Factor I Var})+\beta_{2}(\text{Factor II Var})\\ +\beta_{3}(\text{Factor III Var})\\ +\beta_{4}(\text{Factor IV Var})\\ +\beta_{5}(\text{Factor V Var})+ɛ \end{array}$$

where *ɛ* is a normal distributed random variable with *μ_ɛ_*= 0 and *σ_ɛ_*^2^= *σ*^2^. Similarly, a second regression analysis was carried out to fit the model:

(4) $$\begin{array}{l} \text{Entropy}=\beta_{0}+\beta_{1}(\text{Factor I Mean})\\ +\beta_{2}(\text{Factor II Mean})\\ +\beta_{3}(\text{Factor III Mean})\\ +\beta_{4}(\text{Factor IV Mean})\\ +\beta_{5}(\text{Factor V Mean})+ɛ \end{array}$$

## Results

### Periodicity analyses of entropy profiles

An entropy profile of the aligned 196 bHLH domain sequences (Fig. 1) shows the bHLH domain values have a regular dynamic oscillation. Atchley et al. (2000) suggested the entropy patterns in bHLH correspond to an amphipathic *α*-helix with a variable hydrophilic surface and a conserved hydrophobic surface. Crystal structure studies of individual proteins by Ferre-D’Amare et al. (1993 Ferre-D’Amare et al. (1994) and others show which amino acid sites pack together.

Information in Fig. 1 can be analyzed in a more interpretable manner as a periodogram that describes important features of this pattern. A spectral density plot using the Fourier transformation of the entropy profile has a large peak at approximately 3.77 aa (Fig. 3a). The Burg method result is very similar. Fisher’s test indicates that this periodogram ordinate at 3.77 aa is statistically significant. However, the second and third largest periodogram ordinates are non-significant. Thus, one statistically significant major periodicity component occurs in the entropy profile and it corresponds to the range of known *α*-helix values.

Since the Fourier frequency reported by the FT method gives only an approximate periodicity estimate, harmonic analysis was conducted to detect the best period estimate in the range from 3.30 aa to 3.90 aa, in increments of 0.01. A predictive model was fitted and the associated *R*-square statistics(*R*^2^)calculated for each iteration. The period maximizing the *R*-square statistics has a major periodic component of 3.68 aa repeat with an *R*^2^ = 0.46. A 95% confidence interval of period estimates calculated from 1000 bootstrap entropy profiles includes the value of 3.68. Thus, a major periodic component of bHLH protein domain variability has a periodicity estimate very similar to the conventionally accepted value (3.6 aa per turn) for the elements of ideal individual *α*-helix structure. Thus, the patterns of site by site amino acid variability show a systematic dynamic that is the same as that reported for individual sequences.

Next, we ask if this periodicity pattern can be accounted for by patterns of amino acid physiochemical variability described by Atchley et al. (2005). Multiple regression analyses are carried out where variability of E values are the dependent variables and the factor means or variances are the independent variables.

### Periodicity of Factor Score Means

The factor score means describe the average physiochemical attribute for each amino acid site in each of the five factors (= multidimensional physiochemical attribute index). The spectral density plot of Factor I means (polarity-accessibility-hydrophobicity) is given in Fig. 3b. The peaks located between 3–4 aa suggest possible periodic components and the analysis suggests three possible periodic components of 3.27 aa, 3.77 aa and 2.58 aa. Fisher’s test for the 3.27 aa periodic component is 0.196 (non-significant) but the 3.77 aa component was statistically significant. Thus, there are possible significant periodic components in the Factor I means profile.

The spectral density plots of the means on factors II, III and V (Fig. 3d, 3f, 3i) are not statistically significant. The spectral density plot of Factor IV means (Fig. 3h), on the other hand, has three large periodogram ordinate at 2.58 aa, 3.27 aa, and 4.9 aa. The 2.58 aa component is not significant but the *g* statistic for the 3.27 aa component is significant. Factor IV relates to relative amino acid composition in various proteins, number of codon coding for an amino acid, and amino acid composition.

Multiple regression analysis gave parameter estimates of

$$\begin{array}{l} \beta_{0}=2.931\;(P<.0001),\beta_{1}=-0.082\;(P=0.766),\\ \beta_{2}=-0.045\;(P=0.892),\beta_{3}=-0.380\;(P=0.162),\\ \beta_{4}=-1.086\;(P=0.057)\;\text{and }\beta_{5}=-0.139\\ (P=0.614). \end{array}$$

The proportion of the total variation explained by the model has an *R*^2^ = 0.21 indicating that only 21% of the variation in entropy values could be explained by these five factor score means components. Only factor IV had a regression coefficient approaching statistical significance. Thus, site by site sequence variability is not well explained by the mean factor scores for these multidimensional physiochemical attribute variables.

### Periodicity Analyses of the Factor Score Variances

Next, we explored the relationships between physiochemical variability and the entropy analyses by analyzing the *variances* in factor scores at each site and their relationship to the periodic patterns of variability in the bHLH domain.

An analysis of variance of the factor score variances was statistically highly significant (*P* < 0.0001) indicating that a large amount of the periodic variability could be explained by the variances in the physiochemical attribute factor scores. Hence, we can reject the null hypothesis that there is no difference between the total variation of Factor I–V and the error variance. A multiple regression analysis was carried out of the form:

$$\begin{array}{l} \text{Entropy}=\beta_{0}+\beta_{1}(\text{Factor I Var})+\beta_{2}(\text{Factor II Var})\\ +\beta_{3}(\text{Factor III Var})\\ +\beta_{4}(\text{Factor IV Var})\\ +\beta_{5}(\text{Factor V Var})+ɛ \end{array}$$

Estimates of the regression parameters are:

$$\begin{array}{l} \beta_{0}=0.564\;(P<.0001),\beta_{1}=0.470\;(P=0.052),\\ \beta_{2}=0.468\;(P=0.062),\beta_{3}=0.154\;(P=0.023),\\ \beta_{4}=1.263\;(P<.0001),\beta_{5}=0.174\;(P=0.054). \end{array}$$

The proportion of the total variation explained by the model has an *R*^2^ = 0.86 indicating that 86% of the variation in entropy values could be explained by the variances of these five attribute index variables. Thus, periodic patterns in site by site entropy are strongly related to the amount of variability in these multidimensional physiochemical attribute scores.

The spectral density plot of Factor I variances (Fig. 3c) has peaks at three periodogram ordinates (2.58 aa, 3.77 aa and 3.27 aa) but none are statistically significant in Fisher’s test. However, analyses of the Factor II variances profile give a statistically significant peak at 3.77 aa. A follow-up harmonic analysis gives an accurate period estimate as 3.69 aa (*R*^2^ = 0.285). Similarly, Factor III variances (Fig. 3g) gave a statistically significant peak at 3.77 aa. The follow-up harmonic analysis gives an accurate period estimate as 3.71 aa (*R*^2^ = 0.379). The spectral density plot of Factor IV variances (Fig. 3i) had three peaks at periodogram ordinates at 7 aa, 5.44 aa and 2.13 aa but none are statistically significant. However, the spectral density plot of Factor V variances (Fig. 3k) had large periodogram ordinates at 3.27 aa, 3.77 aa, and 5.44 aa. The value at 3.77 aa is statistically significant in Fisher’s test.

Thus, variability in propensity for secondary structure, molecular size and electrostatic charge are statistically highly significant for predicting patterns of periodicity in site by site amino acid variability. In each instance, the peak occurs at approximately 3.6–3.7 aa, which is close to the conventionally accepted value for an *α*-helix pattern.

## Discussion

Herein, we apply spectral and multivariate statistical analyses to the patterns of amino acid diversity for a broad array of bHLH domain-containing proteins to explore the underlying causes of amino acid diversity. First, we explore the dynamics of amino acid diversity using spectral analysis and then apply regression analyses to account for the underlying causes of periodic sequence diversity by a small set of multidimensional physiochemical indices.

Spectral analyses of site by site sequence variability give periodicity estimates that closely agree with the conventionally accepted value of 3.6 aa for an *α*-helix. Hence, the patterns of amino acid variability for a large sample of aligned proteins closely parallel those seen for the amino acid properties of single proteins.

Are the entropy patterns shown here unique or are they similar to those in other families of proteins with equivalent secondary structure? While periodicity patterns of sequences are not well-known, some data are available. For example, the number of residues per *α*-helical turn in leucine zipper proteins is about 3.64 (Thepaut et al. 2004), a value still very similar to that reported here. Thus, our results for bHLH may reflect a general phenomenon for *α*-helix configurations.

More complicated structural phenomena might affect these estimates. For example, in the basic region of the bHLH protein/DNA complex of the bHLH protein Pho4, there are non-regular *α*-helical turns and the basic region is mostly unfolded relative to residual helical content in the absence of DNA (Cave et al. 2000). Studies on the bHLH-leucine zipper protein Max when uncomplexed with DNA has the first 14 residues of the basic region in a mostly unfolded configuration. However, the last four residues of the basic region form a persistent helical turn while the loop region is observed to be flexible (Sauve et al. 2004). Thus, the various components of the bHLH domain may exhibit different periodicity values depending whether they are complexed with DNA. This is a topic is worthy of further investigation.

One additional concern is whether our removal of part of the highly variable loop region might distort the evaluation of periodicity profiles from multiple alignments. However, we found that removal of part of the loop region had little impact on short-range periodicity. Thus, short-range evaluations, as described here, appear to be robust.

Spectral analysis has a stationarity assumption (Warner, 1998), i.e. the mean and variance of the numeric sequence are constant over amino acid sites and structure depends only on the relative position of two observations (Kendall and Ord, 1990). Thus, it is important to consider the stationarity property of a numeric sequence profile since it can affect the periodicity evaluation. Different regions of a protein sequence may be subject to different regimes of selection during evolutionary divergence and, as a consequence, may display entropy and factor score patterns that are not stationary. In the case of bHLH, partitioning the sequence into several short homogeneous regions and then investigating the periodicity for the basic region, Helix 1 and Helix 2 separately could improve the accuracy of the periodicity evaluation. Such findings are expected because structurally and evolutionarily homogeneous regions intend to be more stationary than the entire sequence.

Several suggestions have been made to deal with the stationarity problem. For example, Warner (1998) has suggested a log transformation of the data might reduce this heterogeneity. Also, complex demodulation methods (e.g. Bloomfield, 1976) make it possible to describe the change in amplitude of the periodic component across amino acid sites more precisely in a non-stationary series.

The analysis of variance and multiple regression approach described here demonstrate that the overall site by site variation (entropy) can be explained by corresponding variation in the major underlying physiochemical attributes of amino acids. By examining the influence of these physiochemical components, we are able to better understand and explain the causes of the observed sequence variability patterns. These results demonstrate that the major periodic components in site by site entropy values and several factor score index variances exhibit the classic *α*-helix periodicity of 3.6 aa. The variances of the factor score for propensity for secondary structure (Factor II), molecular volume (Factor III) and electrostatic charge (Factor V) are significant underlying causal components to site-by-site amino acid diversity in the bHLH domain. Further, the factor score means for polarity and codon composition also contain information related to the helix secondary structure.

These results suggest that periodicity patterns in amino acid diversity reflect significant secondary structure information. Further, entropy as a measure of diversity at each amino acid site can be decomposed into its causal components. As a consequence, these findings should facilitate formal dynamic modeling of both the variability in sequence elements and their underlying causes. Such analyses would provide valuable new information for structural and evolutionary biologists.

Computational techniques, such as applied here, can be powerful estimators of important structural features in proteins. Spectral analysis, in combination with other powerful statistical procedures, can provide valuable information about the periodicities in variability patterns of protein domains, can facilitate other analyses to explore important evolutionary and structural phenomena in proteins, and to significantly enhance our understanding of protein variability, structure, function and evolution. Studies of amino acid variability and periodicity can facilitate protein secondary structure prediction since amino acid variability indeed reflects the underlying structure. Studies similar to this one need to be carried out on protein having different structures to generalize our results.
